# Experimental Study of Bubble Formation from a Micro-Tube in Non-Newtonian Fluid

**DOI:** 10.3390/mi12010071

**Published:** 2021-01-11

**Authors:** Georgia Kontaxi, Yorgos G. Stergiou, Aikaterini A. Mouza

**Affiliations:** Chemical Engineering Department, Aristotle University of Thessaloniki, 54124 Thessaloniki, Greece; gkontaxi@cheng.auth.gr (G.K.); gstergiou@cheng.auth.gr (Y.G.S.)

**Keywords:** microfluidics, bubbles, non-Newtonian fluids, CFD

## Abstract

Over the last few years, microbubbles have found application in biomedicine. In this study, the characteristics of bubbles formed when air is introduced from a micro-tube (internal diameter 110 μm) in non-Newtonian shear thinning fluids are studied. The dependence of the release time and the size of the bubbles on the gas phase rate and liquid phase properties is investigated. The geometrical characteristics of the bubbles are also compared with those formed in Newtonian fluids with similar physical properties. It was found that the final diameter of the bubbles increases by increasing the gas flow rate and the liquid phase viscosity. It was observed that the bubbles formed in a non-Newtonian fluid have practically the same characteristics as those formed in a Newtonian fluid, whose viscosity equals the asymptotic viscosity of the non-Newtonian fluid, leading to the assumption that the shear rate around an under-formation bubble is high, and the viscosity tends to its asymptotic value. To verify this notion, bubble formation was simulated using Computational Fluid Dynamics (CFD). The simulation results revealed that around an under-formation bubble, the shear rate attains a value high enough to lead the viscosity of the non-Newtonian fluid to its asymptotic value.

## 1. Introduction

Bubble technology covers a wide range of academic investigations and industrial applications [1,2,3]. For example, in recent years, the use of microbubbles in biomedical applications, such as molecular imaging, targeted drug delivery, blood oxygenation, treatment of thrombosis, and cancer tumors, has been investigated [4,5,6,7,8]. The size of bubbles used in biomedical applications must be approximately equal to the size of red blood cells, i.e., in the μm scale [1,2,3,4,5,6,7,8,9,10]. Since blood exhibits non-Newtonian behavior, it is necessary to elucidate the mechanism of microbubble formation in order to be able to predict and control their size. It is reported that bubble size can be controlled by altering a variety of parameters such as gas flow rate, viscosity, and surface tension of the liquid phase and micro-tube internal diameter [11,12,13].

Studies concerning bubble formation in both Newtonian, e.g., [13,14] and non-Newtonian, e.g., [12,15,16] fluids are reported in the relevant literature. Some publications concern the bubble formation in microfluidic devices [17,18], while others focus on bubble coalescence [13,19,20]. The formation of bubbles in non-Newtonian fluids in microfluidic devices is still an active field of scientific research and this study can be considered an initial step of a continuing work, whose ultimate goal is to predict and consequently to control the characteristics of bubbles formed from a micro-tube

The scope of the present work is to investigate the characteristics of bubbles formed from a micro-tube in a non-Newtonian fluid. The effect of liquid phase characteristics as well as the gas flow rate is investigated and interpreted.

## 2. Experimental Setup

**Figure 1:**
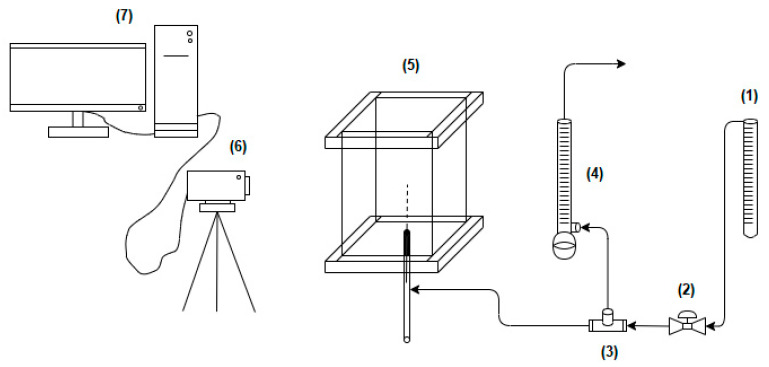
Experimental setup. (1) Gas flowmeter, (2) Valve, (3) Three-way valve, (4), Bubble flowmeter, (5) Plexiglass cell, (6) High-speed digital video camera, and (7) PC.

The experimental setup (Figure 1) consists of a small vertical rectangular PLEXIGLAS cell with a square cross-section of side length 5 cm and height 10 cm. Air was injected through a stainless steel microtube (Hamilton) 110 μm internal.diameter. and 5 cm long installed at the center of the bottom plate. Bubble growth on the tube takes place under constant flow rate. The gas flow rate (0.71 × 10^−6^–1.09 × 10^−6^ m^3^/s) was measured by a bubble flowmeter, while bubble growth was captured by a high-speed video camera (HighSpec4, FASTEC, San Diego, CA, USA).

**Figure 2:**
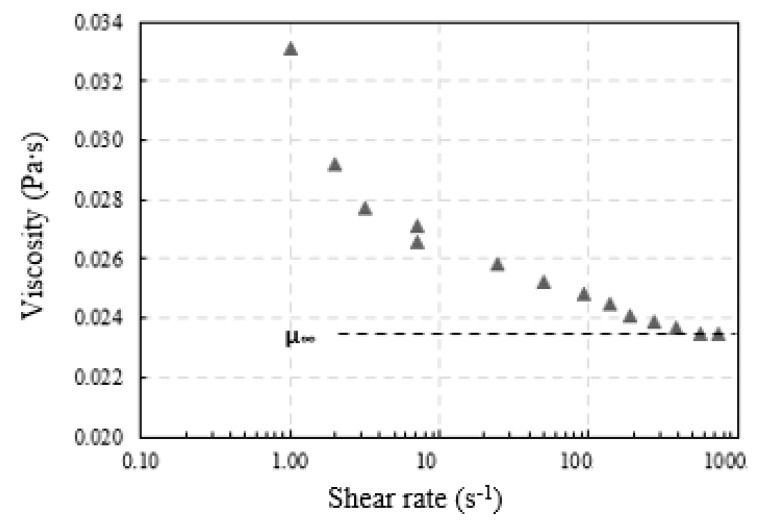
Viscosity versus shear rate for non-Newtonian fluid G2.

All experiments were conducted at ambient pressure and temperature conditions (25 ± 1 °C). The gas phase was air in all cases, while several liquids were used, i.e., distilled water and various Newtonian, aqueous glycerin solutions, and non-Newtonian, glycerin–xanthan gum solutions (Table 1). Xanthan gum is a polysaccharide that renders the solution non-Newtonian. The presence of xanthan gum had a negligible effect on density and surface tension, so that non-Newtonian fluids had the same density and surface tension value as the corresponding Newtonian aqueous solutions. The viscosity of the Newtonian solutions was measured using a KPG-Viscometer, Cannon-Fenske, SCHOTT Instruments GmbH, Mainz, Germany) while the rheological measurements of non-Newtonian fluids were made using a magnetic cone-plate rheometer, type AR-G2 (TA Instruments) (Figure 2). The viscosity of non-Newtonian fluids follows the Herschel–Bulkley model, whose parameters were determined with the magnetic rheometer (Table 1). The surface tension, for both Newtonian and non-Newtonian fluids, was measured with the pendant drop method (OCA 200 CAM, DataPhysics Instruments GmbH, Filderstadt, Germany).

A high-speed digital video camera was employed to capture the various incidents occurring during bubble formation on the tube and thus gain insight into the phenomena. The camera was fixed perpendicularly to the area of observation so that the test section was located between the camera and an appropriate lighting system, which was placed behind a diffuser to evenly distribute the light. During the experiments, the recording rate of the camera was 3000 fps, while the shutter speed was set to 1/1000. By analyzing the recorded images, both the size of the bubbles and the release time (t_0_), i.e., the time required from the moment the bubble is formed on the edge of the tube until it detaches from it, can be calculated.

**Figure 3:**
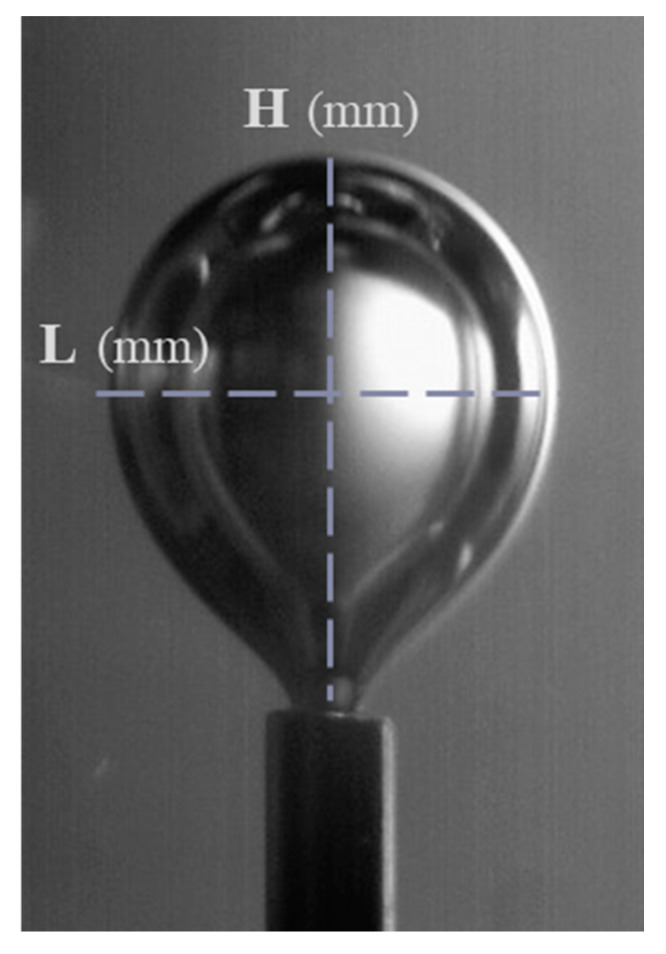
Major (H) and minor (L) axes of the ellipsoid.

The known external diameter of the tube was used for the calibration of the measuring system and the accurate measurement of the bubble size. The final bubble sizes were measured shortly before the detachment moment. The bubbles are approximated by ellipsoids, and the equivalent diameter of a sphere with the same volume as the ellipsoid is computed by the equation:

(1) $$d_{p}=\sqrt[{3}]{H^{2}L}$$

where *d_p_* is the equivalent bubble diameter and *H* and *L* are the major and minor axes of the ellipsoid, respectively (Figure 3). The maximum uncertainty in measuring the length of each axis of the bubble is ± 10 μm and is attributed to the unavoidable shadows formed at the bubble interface. The minimum measured equivalent diameter in the present study was about 4.1 mm, and thus the uncertainty in measuring the size of the bubbles was around ± 8%.

**Figure 4:**
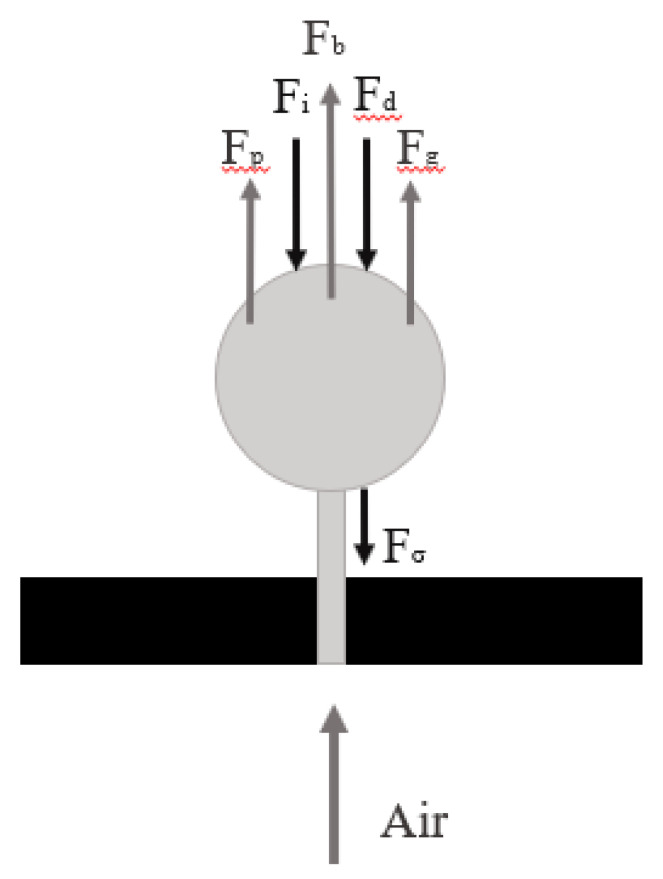
Forces acting on an under-formation bubble.

During the formation stage, a set of forces, which are separated in accordance with their direction, are exerted on the bubble. Therefore, the forces holding the bubble on the μ-tube are the surface tension, the drag force, and the inertia, while the forces that tend to detach the bubble from the μ-tube are momentum force, buoyancy, and pressure (Figure 4). The values of the forces are calculated by Equations (2)–(7) [21].

Surface tension:

(2) $$F_{\sigma }=\pi d_{\alpha }\sigma$$

Drag force:

(3) $$F_{d}=\frac{\pi }{4}d_{p}^{2}C_{d}\rho_{Liquid}\frac{w_{\alpha }^{2}}{2}$$

Inertia force

(4) $$F_{i}=(\alpha +\frac{\rho_{gas}}{\rho_{Liquid}})\rho_{Liquid}Vg$$

where *C_d_* is the average drag coefficient, *w_α_* is the bubble growth rate, α is the bubble growth acceleration, and *d_α_* is the μ-tube diameter.

Momentum force:

(5) $$F_{g}=\frac{\pi }{4}d_{p}^{2}\rho_{gas}w_{g}^{2}$$

Buoyancy force:

(6) $$F_{b}=\frac{\pi }{6}d_{p}^{3}(\rho_{Liquid}-\rho_{gas})g$$

Pressure:

(7) $$F_{p}=\frac{\pi }{4}d_{\alpha }^{2}(P_{g}-P_{L})$$

where *dp* is the equivalent bubble diameter, *w_g_* is the air velocity, *P_g_* is the air pressure in the bubble, and *P_L_* is the average pressure exerted by the liquid.

## 3. Results and Discussion

**Figure 5:**
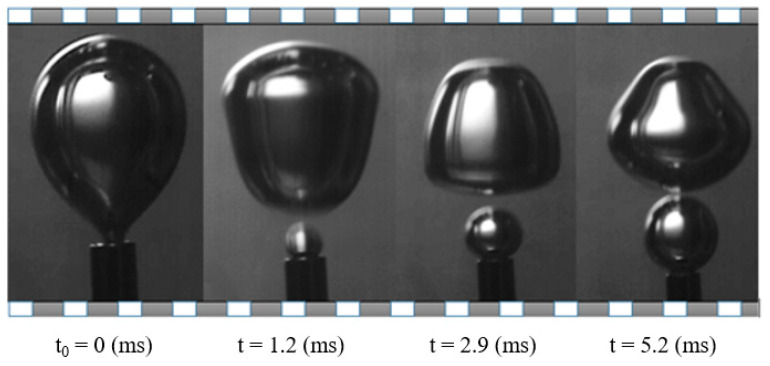
Typical sequence of bubble detachment in non-Newtonian fluid G1 (Q = 1.09 × 10^−6^ m^3^/s).

**Figure 6:**
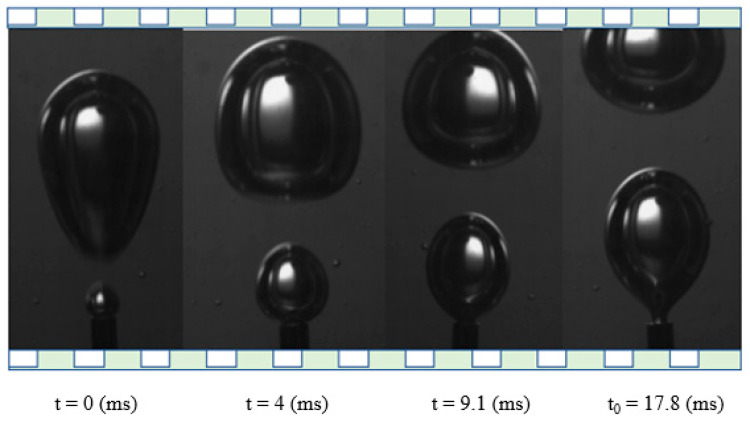
Typical sequence of bubble detachment in non-Newtonian fluid G2 (Q = 1.09 × 10^−6^ m^3^/s).

The experiments in the microscopic scale reveal the effect of gas flow rate as well as type and viscosity of the liquid phase on the bubble characteristics. For the studied flow rates and fluids, no coalescence of the bubbles during the detachment stage was observed. Figure 5 and Figure 6 compare a typical sequence of bubble detachment for the non-Newtonian solution G1 and the non-Newtonian solution G2 respectively for the same flow rate (Q = 1.09 × 10^−6^ m^3^/s). In both cases, the time t_0_ = 0 corresponds to the detachment instant.

### 3.1. Effect of Flow Rate

**Figure 7:**
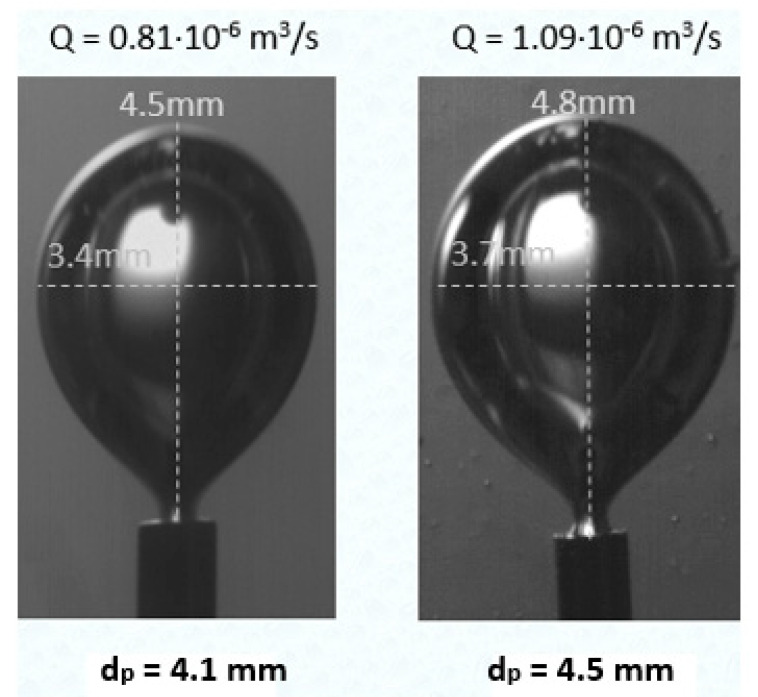
Effect of gas flow rate on bubble size (fluid G1).

For the studied flow rates, it is observed that increasing the flow rate, the bubble equivalent diameter increases in both Newtonian and non-Newtonian fluids (Table 2). Figure 7 shows the increase in bubble size of fluid G1 for flow rates 0.81 × 10^−6^ and 1.09 × 10^−6^ m^3^/s, while it is estimated that a 35% increase in flow rate leads to a 10% increase in the equivalent diameter of the bubble. Similar results are obtained for Newtonian solution G1n for the same flow rates.

Similar results are reported in the relevant literature, e.g., [22,23], which agree with the above experimental observation. However, the complexity of the phenomenon does not allow enough justification for this experimental observation. Increasing the gas flow rate leads to greater gas momentum, faster detachment of the bubble from the tube, and a larger equivalent diameter of the bubble, as shown in Table 1.

The momentum force (Equation (5), Figure 4) is greater, leading to a shorter release time (Table 2). The momentum force depends on the square of the velocity of the gas, but it is weak comparing to all the other forces acting on the bubble during the detachment stage. Specifically, for the minimum flow rate studied (Q_min_ = 0.71 × 10^−6^ m^3^/s), the gas momentum is of the order of 1 × 10^−4^ μN, while for the maximum gas flow rate (Q_max_ = 1.09 × 10^−6^ m^3^/s), the momentum is of the order of 2 × 10^−4^ μN. However, even for the maximum flow rate, the gas momentum remains weak comparing to all the other forces, as the buoyancy is equal to 0.53 N and the pressure is equal to 0.01N.

The increase of the gas flow rate leads to the increase rate of the buoyancy, which translates to rapidly balancing forces exerted on the bubble. Thus, the bubble reaches the detachment point in less time, while its equivalent diameter is larger.

### 3.2. Effect of Viscosity

**Figure 8:**
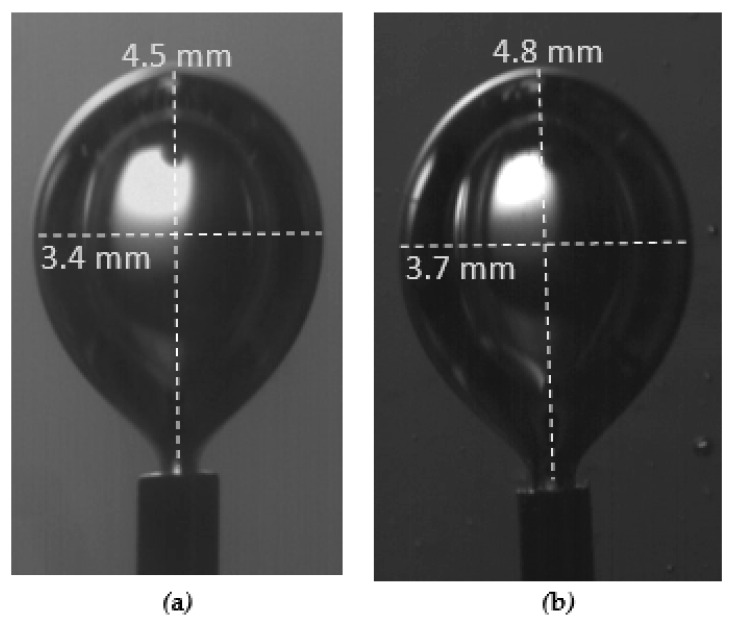
Bubble size comparison during release time t_0_ from the tube for (**a**) G1, (**b**) G2 (Q = 0.81 × 10^−6^ m^3^/s).

All the liquids used, i.e., the Newtonian fluids (G1n, G2n), the non-Newtonian fluids (G1, G2), and water (W), have similar surface tension and density values but considerably different viscosity values (Table 1). It is observed that as the viscosity increases, the release time and the equivalent diameter also increase. Thus, the bubble size increases with increasing asymptotic viscosity (Figure 8). For example, in G1, with an asymptotic viscosity of 8.5 mPa∙s, the equivalent diameter is equal to 4.1 mm, while for G2 with an asymptotic viscosity almost three times greater than that of G1, there is a 5% increase in the equivalent diameter of the bubble. The interpretation of the phenomenon is based on the fact that with higher viscosity, a higher drag force is exerted on the formed bubble, thus delaying its release. These results were obtained under the same flow rate (Q = 0.81 × 10^−6^ m^3^/s).

### 3.3. Effect of the Type of the Liquid Phase

The viscosity of the Newtonian fluids used corresponds to various values of the viscosity curve of the non-Newtonian fluids in order to determine the average viscosity of the fluid around the bubble. Two of the Newtonian fluids used (G1n, G2n) have a viscosity equal to the value of the asymptotic viscosity of the two non-Newtonian fluids (G1, G2).

**Figure 9:**
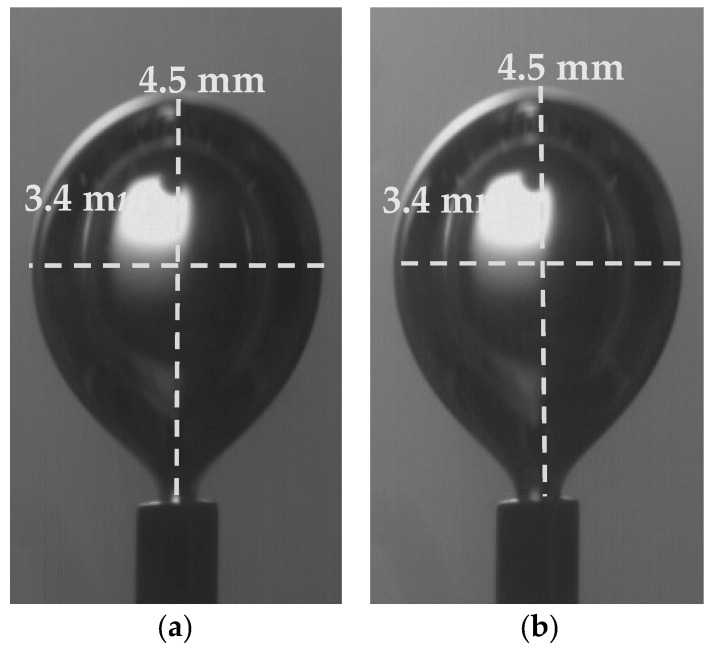
Bubble size during release time t_0_ from tube 110 μm for fluids: (**a**) G1n, (**b**) G1.

It was observed that the bubble size before the release time from the tube in the Newtonian fluid G1n was equal to that of the non-Newtonian fluid whose viscosity is equal to the asymptotic i.e., G1, and the equivalent diameter was 4.1 mm (Figure 9). Respectively, the bubble size in the Newtonian fluid G2n was equal to that of the non-Newtonian fluid G2, and the equivalent diameter was 4.3 mm. The experiments took place under a flow rate of Q = 0.81 × 10^−6^ m^3^/s.

Thus, we can speculate that the shear rate around an under-formation bubble has a high value that in the case of the non-Newtonian fluids corresponds to the asymptotic viscosity value. To confirm this notion, Computational Fluid Dynamics (CFD) simulations were performed.

### 3.4. Computational Study

The simulations were performed using the ANSYS Fluent^®^ R1 code that employs the Volume of Fluid (VOF) method, which is an Euler–Euler approach that is suitable for similar cases [24]. The phenomenon was simulated in two dimensions using axial symmetry. Based on the grid dependence study, a computing space consisting of 306,000 cells was selected. The pressure–velocity coupling was executed using the SIMPLEC algorithm. Respectively, to avoid the effects of the pressure field on the main flow of the bubble, the PRESTO! algorithm was used as the pressure interpolation scheme.

Two experimental cases were simulated, which correspond to the Newtonian fluid G2n (viscosity μ = 23.5 mPa∙s) and the non-Newtonian fluid G2 (asymptotic viscosity μ_∞_ = 23.5 mPa∙s). The boundary conditions correspond to the experimental conditions, and thus the range of the gas velocity at the inlet of the tube equals 1.39—2.24 m/s. A non-slip condition was imposed on the wall of the cell, thus not affecting the bubble formation, while the pressure of the liquid away from the air inlet was considered equal to the atmospheric pressure.

**Figure 10:**
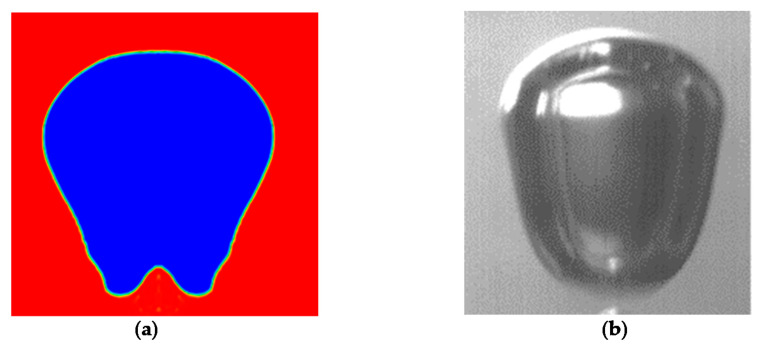
Frame of bubble 1 ms after the detachment: (**a**) Computational Fluid Dynamics (CFD), (**b**) Experiment G2 (Q = 1.09 × 10^−6^ m^3^/s).

The code was validated by comparing the computational results considering the dimensions, the shape, and release time of the bubbles with the ones measured experimentally (Figure 10).

**Figure 11:**
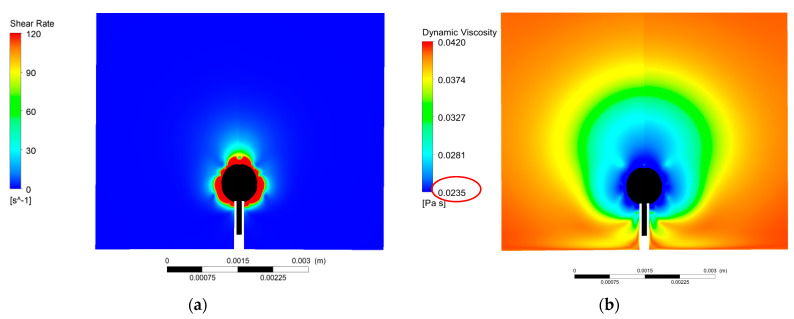
Distribution of (**a**) shear rate and (**b**) viscosity around a formed bubble (G2, Q = 1.09 × 10^−6^ m^3^/s).

The computational results reveal that the shear rate around an under-formation bubble (Figure 11a) corresponds to the asymptotic viscosity (Figure 11b) of the G2 liquid (Table 1). Around the surface area of the bubble the viscosity attains its lower value, which is equal to the asymptotic viscosity μ_∞_ = 23.5 mPa∙s, while away from the bubble surface, the shear rate decreases, leading to lower viscosity values. This result interprets the experimental finding, i.e., that the bubbles formed in a non-Newtonian fluid have practically the same equivalent diameter with the bubbles formed in a Newtonian fluid with viscosity equal to the asymptotic viscosity of the non-Newtonian fluid.

## 4. Conclusions

Experiments in the micro-scale were conducted for Newtonian and non-Newtonian fluids. The results enhance our comprehension of the bubble behavior in non-Newtonian fluids. From the liquids tested, it was found that by increasing the flow rate (Q = 0.71 × 10^−6^–1.09 × 10^−6^ m^3^/s) the bubble reaches the detachment point in less time, while its equivalent diameter is larger. As the viscosity increases, the release time and the equivalent diameter also increase. Thus, the bubble size increases with increasing asymptotic viscosity, while around the bubble, the shear rate is high, and as a result, the viscosity around the bubble in a non-Newtonian solution has a value equal to that of asymptotic viscosity. The bubbles formed in Newtonian fluid with viscosity equal to the asymptotic viscosity of the non-Newtonian have the same equivalent diameter with the bubbles formed in the non-Newtonian fluid. The bubble behavior is strongly affected by the gas flow rate and the viscosity. Future study on the effect of the other parameters would lead to an even better interpretation of the phenomenon.

## Figures and Tables

**Table 1:** Properties of the studied Newtonian and non-Newtonian fluids.

Liquid Phase	Content			*ρ* (kg/m^3^)	*μ* (mPa∙s)	*μ_∞_* (mPa∙s)	*σ* (mN/m)
	Water (%*v*/*v*)	Glycerin (%*v*/*v*)	Xanthan (g/100 mL)				
W	100	-	-	997	1	-	72
G1n	45	55	-	1140	8.5	-	68
G1	50	50	0.025	1126	0.0332 + 0.0421γ^0.75^	8.5	68
G2n	28	72	-	1186	23.5	-	67
G2	30	70	0.025	1181	0.0098 + 0.0271γ^0.98^	23.5	67

**Table 2:** Dependence of bubble size and release time on gas flow rate for G1.

Flow Rate Q (m^3^/s)	Equivalent Diameter *d_p_* (mm)	Release Time *t* (s)	Bubble Volume V (10^−8^ m^3^)
0.71	3.9	0.027	1.92
0.81	4.1	0.017	1.38
0.94	4.4	0.015	1.41
1.09	4.5	0.013	1.42
